# A Precise, Controllable *in vitro* Model for Diffuse Axonal Injury Through Uniaxial Stretch Injury

**DOI:** 10.3389/fnins.2019.01063

**Published:** 2019-10-17

**Authors:** Yu Li, Chaoxi Li, Chao Gan, Kai Zhao, Jianbin Chen, Jinning Song, Ting Lei

**Affiliations:** ^1^Department of Neurosurgery, Tongji Hospital, Tongji Medical College, Huazhong University of Science and Technology, Wuhan, China; ^2^Department of Neurosurgery, The First Affiliated Hospital of Xi’an Jiaotong University, Xi’an Jiaotong University, Xi’an, China

**Keywords:** traumatic brain injury (TBI), diffuse axonal injury (DAI), traumatic axonal injury (TAI), stretch, *in vitro* model

## Abstract

Regarding the determination of the biomechanical parameters in a reliable *in vitro* cell model for diffuse axonal injury (DAI), our study aimed to demonstrate connections between those parameters and secondary axotomy through examination of morphological alterations under a variety of traumatic conditions. An *in vitro* cell model for DAI was established in primary cultured mouse neurons by uniaxial mechanical stretching of non-myelinated axons under various traumatic conditions: strain (ε) = 5, 10, 20, and 50%; strain time (*t*) = 500, 100, and 20 ms; strain rate ranging between 0.1 and 25 s^–1^. Axonal real strains (strain_axon_) were measured as 4.53 ± 0.27, 9.02 ± 0.91, 17.75 ± 1.65, and 41.8 ± 4.4%. Axonal real strain rates (SR_axon_) ranged between 0.096 ± 0.0054 and 20.9 ± 2.2 s^–1^. Results showed there was no obvious abnormality of axons with a lower strain condition (strain_axon_ < 17.75 ± 1.65%) during the acute phase within 30 min after injury. In contrast, acute axonal degeneration (AAD) was observed in the axons following injury with a higher strain condition (SR_axon_ > 17.75 ± 1.65%). In addition, the incidence and degree of AAD were closely correlated with strain rate. Specifically, AAD occurred to all axons that were examined, when ε = 50% (strain_axon_ = 41.8 ± 4.4%) for 20 ms, while no spontaneous rupture was observed in those axons. Besides, the concentration of Ca^2+^ within the axonal process was significantly increased under such traumatic conditions. Moreover, the continuity of axon cytoskeleton was interrupted, eventually resulting in neuronal death during subacute stage following injury. In this study, we found that there is a minimum strain threshold for the occurrence of AAD in non-myelinated axons of primary cultured mouse neurons, which ranges between 9.02 ± 0.91 and 17.75 ± 1.65%. Basically, the severity of axonal secondary axotomy post DAI is strain rate dependent under a higher strain above the threshold. Hence, a reliable and reproducible *in vitro* cell model for DAI was established, when ε = 50% (strain_axon_ = 41.8 ± 4.4%) for 20 ms.

## Introduction

According to an estimation model based on previous epidemiological studies, traumatic brain injury (TBI) is a major cause of global mortality (Hyder et al., 2007; Rubiano et al., 2015). Diffuse axonal injury (DAI), one of the most important pathological subtypes of TBI, occurs in 50% of admitted patients who have suffered TBI (Meythaler et al., 2001). In cases of DAI, many axons become overstretched because they have sustained tremendously accelerated motion caused by overwhelming forces during unwanted incidents such as car crashes and sport accidents (Povlishock and Christman, 1995). This inevitably results in axonal degeneration during the phase of secondary axotomy. This phase is characterized by focal axonal beads and secondary disconnection that can also lead to the extensive shutdown of neural networks and long-term coma in patients.

Since the first report of DAI by Strich (1956), we have made tremendous progress in understanding the mechanisms underlying this condition. Furthermore, the methods used in clinical diagnosis have also advanced, largely through the development of cutting-edge medical imaging technologies and a better understanding of DAI itself. Nevertheless, our knowledge of how to treat DAI remains insufficient, and the rates of mortality and morbidity of patients with DAI are extremely poor (Smith et al., 2013). One obstacle that has yet to be overcome is to establish a reliable and stable preclinical model for DAI that can be used to determine the precise mechanism involved and also identify effective treatments. Early experiments featuring *in vivo* animal models of DAI involved fixing the heads of animals on a customized rotating device, through which violent rotation was used to cause a subsequent trauma. Previous studies had initially applied these techniques to the skulls of large mammals such as baboons and pigs (Gennarelli et al., 1982; Ross et al., 1994) and were subsequently applied to rats using a modified device (He et al., 2004; Wang et al., 2010; Li et al., 2013). Marmarou et al. (1994) also established a rat copper-rod strike model, which was relatively easy to construct, and allowed significant progress in the *in vivo* modeling of DAI.

Considering the evident variation associated with animal models, one reasonable strategy is to develop a simple cell model for DAI under relatively simple, enclosed, and controllable conditions. For example, Smith et al. (1999) proposed an *in vitro* DAI model in which cultured neurons were forced to overstretch (ε = 0.58–0.77) by using a controlled air pulse for 26 − 35 ms. Staal et al. (2007), however, reported a modified form of overstretching (ε = 0.02 − 0.1), which was induced by controlled shear stress injury with a 20-ms time duration. In another study, LaPlaca et al. (2005) established an updated model (ε = 0.25–0.5), which induced DAI by mechanical stretching over a 13–20-ms period. Kilinc et al. (2009) also described a method to induce DAI using shear stress injury (45 dyn/cm^2^); mean onset time was approximately 20 ms (Dollé, 2013). In summary, these previous studies collectively revealed that there are significant strain-related variations among different DAI models, even though onset times were also reported to be approximately 20 ms.

This study aimed to develop a standard, but novel, *in vitro* cell model of DAI. To achieve this, axons were overstretched in primary cultured mouse neurons using a uniaxial mechanical stretching technique under a variety of traumatic conditions involving different degrees of strains and strain rates. Subsequently, the actual values of these parameters (strain and strain rate), as well as morphological alterations, were detected in axons under various designated circumstances during the acute phase following DAI injury, thereby establishing a baseline for a new, standard *in vitro* cell model of DAI.

## Materials and Methods

### Cortical Neuronal Cultures

Primary cortical neurons were isolated from male QSi5 mice on postnatal day 0 (P0) as described previously (Beaudoin et al., 2012). In brief, mice were first decapitated after being sterilized with 75% ethanol. Cerebral tissues were then transferred into ice-cold D-hanks buffer, and the meninges were removed in their entirety. The entire cerebral cortex was then dissected from the brain, chopped into smaller pieces, digested with 0.25% trypsin (T4424, Sigma-Aldrich, St. Louis, MO, United States) for 15 min and then in 0.1% DNase (DN25, Sigma-Aldrich, St. Louis, MO, United States) for another 5 min in a 37°C water bath. After being washed twice with planting medium containing 88% Dulbecco’s modified eagle medium (DMEM, 12491-015, Gibco, Thermo Fisher Scientific, Rochester, NY, United States), 10% fetal bovine serum (FBS, 16140071, Thermo Fisher Scientific, Rochester, NY, United States), 1% glutamine (25030-081, Gibco, Thermo Fisher Scientific, Rochester, NY, United States), and 1% penicillin/streptomycin (15140-122, Gibco, Thermo Fisher Scientific, Rochester, NY, United States), tissues were gently triturated eight times using a sterile glass pipette. Cell solutions were then centrifuged at 200 × *g* for 30 s, and the supernatant was retained to transfer either into elastic chambers (ST-CH-04, B-Bridge International, Inc., Sunnyvale, CA, United States) or onto coverslips (Sigma-Aldrich, St. Louis, MO, United States), both of which were pretreated with 0.5 mg/ml poly-L-lysine (P-9155, Sigma-Aldrich, St. Louis, MO, United States). Cell culture experiments in the elastic chambers involved a 2 mm × 20-mm silicone barrier made with Sylgard 184 silicone elastomer, which was located in the middle of the elastic chamber with cultured neurons, creating an axon-only area. Cells were allowed to attach for 24 h before the barrier was removed. Following barrier removal, axons traversed, ultimately integrating with neurons on the other side (Supplementary Figure S1). Six hours after cell plating, the culture medium was refreshed by long-term maintenance medium, half of which was regularly replaced every other day. At 48–72 h post-planting, cytarabine (final concentration, 4 μM; C3350000, Sigma-Aldrich, St. Louis, MO, United States) was added into the maintenance medium to suppress the proliferation of non-neuronal cells. These experiments were performed *in vitro* for 7 − 12 days (Supplementary Figure S2).

### Identification of Cell Cultures

Due to the lower capability for dendritic outgrowth, compared with axons, the central region of 2 mm × 20-mm silicone barrier area normally contained axons only. Microtubule-associated protein 2 (MAP2, a specific marker for dendrites and neuronal somata) and microtubule-associated protein Tau (a specific marker for axons and neuronal somata) were used to identify neurites in the axon-only area. Cells were labeled with either monoclonal mouse anti-MAP2 antibody (1:2,000; clone HM-2, Sigma-Aldrich, St. Louis, MO, United States) or Tau (1:1,000; clone Tau46, Sigma-Aldrich, St. Louis, MO, United States), followed by Alexa Fluor 594 goat anti-mouse IgG1 (1:100 Molecular Probes, Thermo Fisher Scientific, Rochester, NY, United States). Detailed methods are described below in the section relating to immunocytochemistry. To test the purity of our neuronal cell cultures, cultured cells were double labeled with polyclonal rabbit anti-β-tubulin antibody (1:500, Cell Signaling, Danvers, MA, United States) and a neuronal cell maker (either MAP2 or Tau). Alexa Fluor 594 goat anti-rabbit IgG and Alexa Fluor 488 goat anti-mouse IgG were used as corresponding secondary antibodies. A high ratio of MAP2 to Tau-positive cells compared to β-tubulin III-positive cells demonstrated the purity of the cultured neurons.

### Axonal Stretch and Strain Analysis

As mentioned above, primary neurons cultured in silicone chambers were fixed into a modified STREX ST-150 strain instrument (B-Bridge International, Inc., Sunnyvale, CA, United States), a motor-driven instrument that deforms silicone chambers to stretch cultured neurons. The motor can be replaced to adjust the extent of stretch by rotation at different velocities. The silicone chambers were continuously perfused with pre-warmed solution (1.5 ml/min) at a preselected temperature of 36–37°C. The perfusion tube was fixed at the upper right side, and a suction needle connected with the vacuum aspiration device was fixed at the lower left side of the chamber. In order to minimize the shear stress injury caused by fluid as much as possible, the distance of the suction needle was not less than 7 mm away from the bottom of the silica gel Petri dish (the Petri dish depth is 1 cm).

In this experiment, a single uniaxial stretch of 1, 2, 4, and 10 mm was applied to the chamber within durations of 500, 100, and 20 ms, respectively. We then restored the initial state. Single axons of diameters ranging from 0.75 to 1.25 μm, with a deviation of less than 30° from the *x*-axis (the stretch direction), were included for stretch experiments. Given the non-rigid connection between cultured neurons and the chambers, maximal axonal strains were determined by actual measurements when the chambers reached their maximum deformation. Before stretching, axonal lengths were measured as *L*_0_. The silicone chamber was then stretched and was held still at a certain stretching state to measure axonal lengths, defined as *L*_max_. Axonal real strain (ε) was repeatedly measured in each group (*n* = 6) using the following formula: ε = (*L*_max_-*L*_0_)/*L*_0_. Strain rate (*´ε*) was measured as follows: *´ε* = dε/d*t* (*n* = 6).

Since the stretch-induced strain field distribution on the cell culture area of the silicone chamber may not have been uniform, we performed simulations using the finite element analysis (FEA) software ANSYS 14.0 under distinct conditions (4 and 10 mm stretch). The silicone chamber was simulated using cubic three-dimensional (3-D) elements. A rigid cylinder was established to stretch the circular hole of the Petri dish with corresponding mechanical parameters. To simulate chamber deformation, we performed a non-linear, large-deformation static analysis to determine the strain field distribution when the maximum stretch is reached. For all simulations, we used a Young modulus of 2.5 MPa and a Poisson ratio of 0.45 (B-Bridge, Santa Clara, CA, United States). In order to ensure that axons underwent the same extent of stretching, we only selected neurons located within the center area (2 mm width × 2 mm length), also known as the axon-only region for further analysis.

### Morphological Analysis of Axons

Stretch-induced acute axonal degeneration AAD mostly manifests as beading (localized swelling), with or without secondary disconnection. In this study, a localized swollen area along the axon was defined as a bead in which the diameter was at least twice that of the adjacent areas. Transmitted light images were captured under confocal microscopy (Leica Microsystems, Buffalo Grove, IL, United States) for the same axon before stretch and 30 s and 2, 5, 10, and 30 min post-stretch. ImageJ software (NIH, Bethesda, MD, United States) was used to count beads. Sham and stretched axon preparations were processed and analyzed in parallel under the same experimental conditions except for the stretching.

### Cell Death

At acute and subacute stages, post-stretch (1 and 24 h post-stretch, respectively) cell viability was investigated using propidium iodide (PI; Sigma-Aldrich, St. Louis, MO, United States), which labels dead or dying cells. Cells were washed once with pre-warmed artificial cerebral spinal fluid (ACSF, 137 mM NaCl, 5 mM KCl, 5.6 mM glucose, 20 mM HEPES, 0.6 mM KH_2_PO_4_, 0.5 mM Na_2_HPO_4_, 2.0 mM CaCl_2_, and 1.0 mM MgCl_2_) and then incubated with 50 μg/ml of PI for 15 min at 37°C. Dead or dying cells were recorded by fluorescent images (λ_ex_: 540 nm; λ_em_: 608 nm), and the total number of cells was recorded from transmitted light images. For each chamber, images were captured at 12 adjacent microscopic fields along both sides of the axon-only regions. The ratio of cell death was calculated as the number of PI-positive cells against total cell numbers. Moreover, 24 h after culturing, a positive control for PI staining (5 μg/ml) was applied as treatment with 0.5 mM glutamate (Sigma-Aldrich, St. Louis, MO, United States) for 30 min.

### Ca^2+^ Imaging

Fluo-4 AM fluorescent dye (Molecular Probes, Thermo Fisher Scientific, Rochester, NY, United States) was used for intracellular Ca^2+^ measurements. Cells were washed once with pre-warmed ACSF and then incubated with 2.5 μM fluo-4 AM for 20 min at 37°C. Then, cells were rinsed in order to incubate with ACSF for another 20 min, in turn allowing further de-esterification of the dye. Images were captured under confocal microscopy (Leica Microsystems, Buffalo Grove, IL, United States). The excitatory wavelength was 488 nm, and emissions were collected in the 505–515 nm range. To account for potential variation in dye loading among axons or experiments, a standard procedure was applied with non-ratiometric indicators, in which self-ratios were taken (*F*/*F*_0_) between the measured fluorescence (*F*) and the initial fluorescence (*F*_0_). Background fluorescence subtraction was accomplished by continuously sampling three areas in the field that had no axons in them for the duration of the experiment. Since fluo-4 AM is a non-ratiometric dye, we used CellTracker Red (CTR), a cytoplasmic marker, as a reference dye to avoid volumetric effects. In these experiments, 2.5 μM fluo-4 AM was used in tandem with 3 μM CTR. For CTR, the excitatory wavelength was 594 nm, and emissions were collected within the range of 600–610 nm.

### Immunocytochemistry

Cells were fixed in 4% pre-warmed (37°C) paraformaldehyde for 15 min, and 0.1% Triton was used to permeabilize cell membranes for 5 min at room temperature. Then, 10% goat serum (GS; Thermo Fisher Scientific, Rochester, NY, United States) with 1% bovine serum albumin (BSA; Sigma-Aldrich, St. Louis, MO, United States) in phosphate-buffered saline (PBS) was used to block non-specific sites for 1 h at room temperature. Cells were then incubated with primary antibodies overnight in a humidified chamber at 4°C. The primary antibodies used for immunocytochemistry were as follows: monoclonal mouse anti-MAP2 antibody (1:2,000; clone HM-2, Sigma-Aldrich, St. Louis, MO, United States) or Tau (1:1,000; clone Tau46, Sigma-Aldrich, St. Louis, MO, United States) and polyclonal rabbit anti-β-tubulin III antibody (1:1,000, Sigma-Aldrich, St. Louis, MO, United States). After being washed three times with PBS, cells were incubated for 2 h with fluorescent dye-conjugated secondary antibodies, as follows: Alexa Fluor 488 goat anti-mouse IgG2a (1:100; Molecular Probes, Thermo Fisher Scientific, Rochester, NY, United States); Alexa Fluor 594 goat anti-rabbit IgG (1:100; Molecular Probes, Thermo Fisher Scientific, Rochester, NY, United States); Alexa Fluor 594 goat anti-mouse IgG (1:100; Molecular Probes, Thermo Fisher Scientific, Rochester, NY, United States). All antibodies were diluted with 5% GS and 0.5% BSA in PBS. Finally, cells were mounted in ProLong Gold antifade reagent with 4′,6-diamidino-2-phenylindole (DAPI; Thermo Fisher Scientific, Rochester, NY, United States), the latter being a water-soluble nuclear and chromosomal counterstain. Immunofluorescence was examined under a Zeiss LSM 510 Meta confocal microscope (Carl-Zeiss, Jena, Germany). Images were processed with ImageJ software (NIH, Bethesda, MD, United States). For negative controls, cells were incubated with secondary antibodies only.

## Results

### FEA Strain Field Analysis and Axon Actual Strain Measurement

**FIGURE 1:**
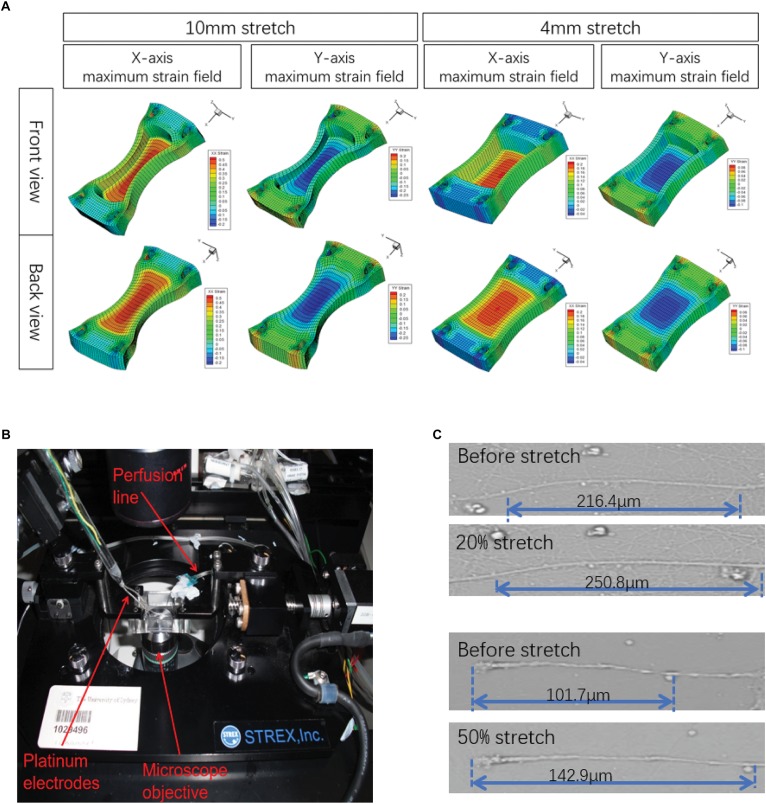
Illustration of the experimental design. **(A)** A stretch unit (STREX ST-150) as visualized by confocal microscopy. **(B)** Finite element analysis (FEA) was simulated in ANSYS 14.5 software to model the strain field distribution in the silicone chamber. Only the central areas (2 mm × 2 mm) of the axon-only region were selected for further analysis. **(C)** Actual maximum strains (ε) of axons were calculated by axonal length measurement before and after stretch, respectively.

First, in order to ensure that the axons in this study were stretched with deviation as small as possible, the strain fields of Petri dishes under the maximum deformation under different parameters were tested by FEA (Figure 1A). The results showed that the strain field of the Petri dish in the pull direction (*x*-axis) was terraced, and the maximum strain was obtained in the central region of the Petri dish, which was 0.05, 0.1, 0.2, and 0.5. At the same time, a negative strain field (compression) was formed in the *y*-axis direction when tension was carried out along the *x*-axis. The maximum negative strain in the central region was −0.025, −0.05, −0.1, and −0.25. The above results indicated that the axons in the 2 mm × 2 mm area of the Petri dish center selected in this study can theoretically achieve the largest as well as relatively homogeneous biomechanical effects. In addition, the angle between axon projection and *x*-axis was found to be less than 30°, so the main injury effect of axons is *x*-axis tension rather than *y*-axis compression.

Although the axons attach to the bottom of the Petri dish, their connections were basically not rigid. Therefore, under intense strain conditions, because of the relative displacement between the Petri dish and axons, the strain that they obtain may not be the same when it occurs. Therefore, we sat the stretch-hold mode (stretch-release mode) to study axonal real strain (ε) under maximum stretch (Figure 1C). The results showed that there was a real strain along the *x*-axis of the axon, and the actual strain was lower than the Table 1 simulated by FEA.

**TABLE 1:** Parameters under distinct stretch conditions.

Stretch distance (mm)	1			2			4			10		
Strain (chamber)	5%			10%			20%			50%		
Strain (axon)	4.53 ± 0.27%			9.02 ± 0.91%			17.75 ± 1.65%			41.84 ± 4.4%		
Time cause (ms)	500	100	20	500	100	20	500	100	20	500	100	20
Strain rate (chamber) (s^–1^)	0.1	0.5	2.5	0.2	1	5	0.4	2	10	1	5	25
Strain rate (axon) (s^–1^)	0.096 ±	0.48 ±	2.4 ±	0.18 ±	0.92 ±	4.6 ±	0.355 ±	1.775 ±	8.875 ±	0.836 ±	4.18 ±	20.9 ±
	0.0054	0.027	0.135	0.0182	0.091	0.455	0.033	0.165	0.825	0.084	0.44	2.2

Based on that, primary neurons cultured in silicone chambers were fixed into a modified STREX ST-150 strain instrument and precisely controllable for the extent of stretch by rotation at different velocities with constant perfusion (1.5 ml/min) of a pre-warmed solution at 36–37°C (Figure 1A). In this experiment, a single uniaxial stretch of 1, 2, 4, and 10 mm was applied to the chamber within durations of 500, 100, and 20 ms (Figure 1B). First, the initial state was recorded. Then, the silicone chamber was stretched. Axonal real strain (ε), as well as strain rate (*´ε*), were repeatedly measured in each group (*n* = 6) (Figure 1C). The parameters used in different experimental groups are shown in Table 1. Our results revealed varied distributions of strain fields (Figure 1B). In order to ensure that axons underwent identical stretches, we only selected those axons located within the center area (2 mm width × 2 mm length), also known as the axon-only region for further analysis.

### Compartmentalization Culture of Axons and Somata

**FIGURE 2:**
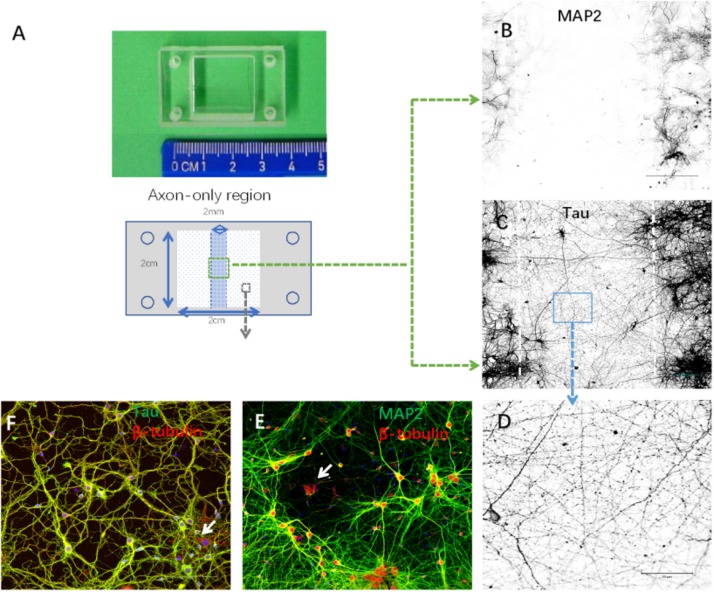
Establishment of cortical neuron cultures in a silicon chamber. **(A)** Silicon chamber (STREX) for primary neuron culture, upper. From the back view of the device, schematic illustration of the axon-only area (in the middle area with shadowing, 2 mm width × 2 cm length) and neuronal cell body region (white square area with spots) below. **(B–D)** Neurites in the axon-only area were axons confirmed by Tau-positive **(C)** but MAP2-negative **(B)** immunofluorescence. Representative image **(D)** of Tau immunostaining from the boxed area **(C)** shown under higher magnification. **(E,F)** Neurites in the somata region with positive immunoreactivity to either MAP2 **(E)** or Tau **(F)**. Only a few non-neuronal cells [arrows in **(E)**, β-tubulin-positive and MAP2-negative; arrows in **(F)**, β-tubulin positive and Tau negative] were evident in the primary cortical neuron cultures. Scale bar = 100 μm.

In order to isolate axons without compromising their structural integrity, we set up compartmentalized cultures of axons and somata in silicone chambers (Figure 2A). Neurites in axon-only regions showed an absence of MAP2 and the presence of Tau immunoreactivity (Figures 2B–D), suggesting that this region consisted only of axons. In contrast, most cells exhibited MAP2-, Tau-, and β-tubulin-positive staining in somata regions. Meanwhile, a small portion of cells (<10%, data not shown) exhibited β-tubulin-positive but MAP2/Tau-negative staining. In summary, these results demonstrated that we purified a high yield of neurons (Figures 2E,F).

### Minimum Threshold Strain for AAD and Strain Rate-Dependent AAD

**FIGURE 3:**
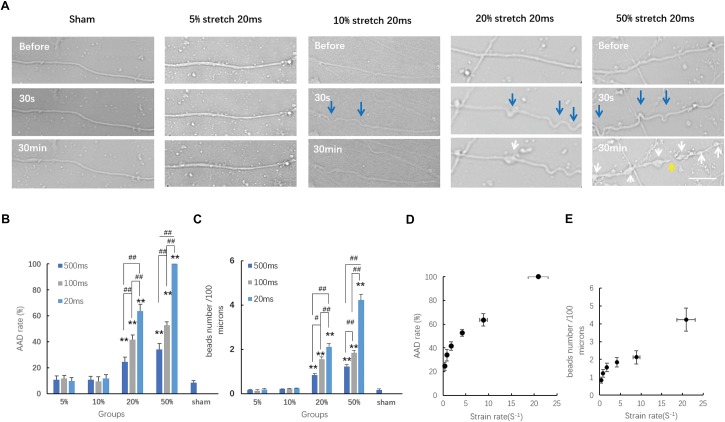
Axon responses to stretch-induced injury during the chronic phase. **(A)** Representative serial images of axonal responses after being subjected to distinct conditions for 20 ms using transmitted light under confocal microscopy. Blue arrows indicate the undulations exhibited immediately after stretch. White arrows indicate the formation of beads. The yellow arrow indicates the secondary disconnection that occurred in axons within 30 min after stretch. Scale bar = 20 μm. Quantification of the incidence of acute axonal degeneration (AAD) **(B)** and the extent of AAD **(C)** in different groups. Analyzed by Kruskal–Wallis test and *post hoc* Mann–Whitney *U*-test. ^##^*P* < 0.01; ^∗∗^*P* < 0.01 (compared with the sham group). **(D)** Plot of AAD occurrence rate at 30 min post-injury change over strain rates; logistic regression analysis; *P* = 0.026. **(E)** Plot of the number of beads per 100 μm length of axon at 30 min post-injury change over stain rates; analyzed by logistic regression analysis; *P* < 0.01; all data were presented as mean ± SE.

Within the acute period (30 min) after stretch injury, we detected morphological changes in the axons under transmitted light images before stretch and 30 s–30 min after stretch (Figure 3A). Most sham-injured single axons, as expected, exhibited a normal axonal appearance from the beginning to the end (Figure 3A). Moreover, even axons that underwent 5 and 10% stretch for durations of 500, 100, and 20 ms exhibited no detectable alterations in morphology (AAD rates and bead number, compared with sham, all *P* > 0.05, Figures 3B,C). For single axons that underwent 20 and 50% stretch, we observed clear undulations immediately after stretch. Localized swelling then started to emerge and gradually enlarged over time, eventually leading to bead formation (Figure 3A). When the strain was greater than 20%, stretch-induced AAD was significantly increased, compared with that in the sham group (Figures 3B,C). These results demonstrated that the minimum threshold strain for stretch-induced AAD was between 10 and 20% (real axonal strain, 9.02 ± 0.91 to 17.75 ± 1.65%).

We also analyzed the effects of different strain rates upon stretch-induced AAD. Our results showed that when the strain was larger than the minimum threshold (20 and 50%), then this higher strain rate caused more severe AAD (Figures 3D,E). This indicated that the degree of stretch-induced axon degeneration was potentially strain rate dependent. For axons treated with a strain smaller than the minimum threshold (10% strain, *t* = 100 and 20 ms), even though the strain rates subjected to these axons were significantly higher than those in the groups with 20% strain lasting for either 500 or 100 ms, respectively (*P* < 0.01), they produced a lower incidence of axon degeneration compared with that in the same groups (*P* < 0.01). These results suggested that AAD was strain rate dependent only when the minimum strain threshold was exceeded.

### Calcium Dynamics in Injured Axons

**FIGURE 4:**
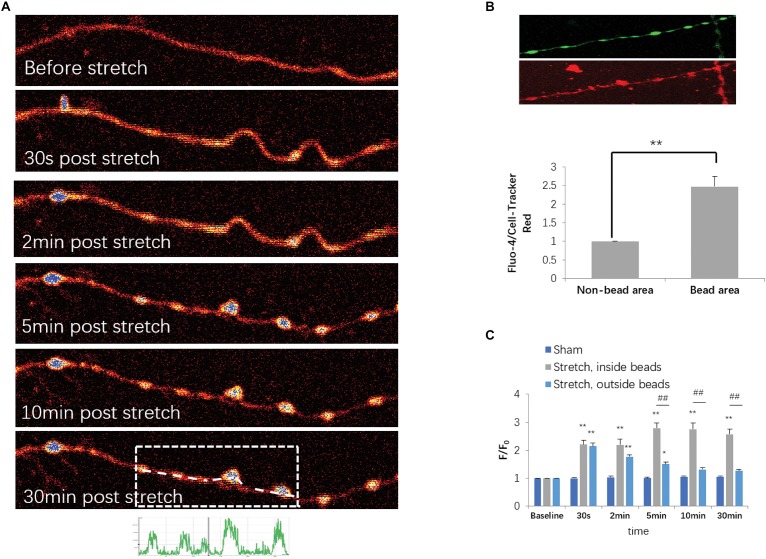
Stretch-triggered [Ca^2+^]_axo_ dynamic alterations. **(A)** Representative serial images of fluo-4-loaded axons with pseudocolor at designated time points. **(B)** Upper panel, fluo-4- and CellTracker Red (CTR)-labeled axons at 30 min after stretch. Lower panel, fluo-4 intensities normalized against CTR intensities for comparison of [Ca^2+^]_axo_ between the sites within and outside of the spheroids (paired *t-*test, ^∗∗^*P* < 0.01; *n* = 7). **(C)** Quantitative analysis for [Ca^2+^]_axo_ in neurons subjected to either sham operation or stretch for each duration range (all data were presented as mean ± SE; one-way ANOVA, *n* ≥ 6; ^∗∗^*P* < 0.01, comparison between sham groups and stretch inside the spheroids groups; ^#^*P* < 0.05, ^##^*P* < 0.01, comparisons between sham groups and stretch outside of the spheroid groups; ^&^*P* < 0.05, ^&&^*P* < 0.01, comparisons between stretch within and out of the spheroid groups).

Based on the fact that an increase in axoplasmic Ca2+ ([Ca^2+^]_axo_) is a key cellular event for stretch-induced AAD, we also investigated stretch injury-induced dynamic [Ca^2+^]_axo_ change in our model using confocal calcium imaging and fluo-4 AM. Because a 50% stretch for 20 ms resulted in a much higher occurrence rate of axon degeneration than that in the other groups, only those neurons that were subjected to 50% stretch for 20 ms were selected for the determination of [Ca^2+^]_axo_. As previously reported, [Ca^2+^]_axo_ increased significantly following mechanical stretch. Specifically, from 2 to 30 min after stretch, this increase was even more notable in the beading areas compared with that in the non-beading areas (Figures 4A,B). To eliminate the artifacts arising from volume change, fluo-4 intensities were normalized against the intensities of CTR in order to compare [Ca^2+^]_axo_ between the beading areas and non-beading areas. We found that the fluo-4/CTR ratio of beading areas was significantly higher than that of non-beading areas (Figure 4C). Non-beading areas only manifested as a transient [Ca^2+^]_axo_ increase, which lasted approximately 10 to 30 min after stretch. However, axonal beading areas showed a higher and more persistent increase in Ca^2+^ influx ([Ca^2+^]*_i_*), suggesting that distinct mechanisms of [Ca^2+^] increase may be involved in these two axonal areas after stretch injury.

### Cytoskeletal Damage in Injured Axons

**FIGURE 5:**
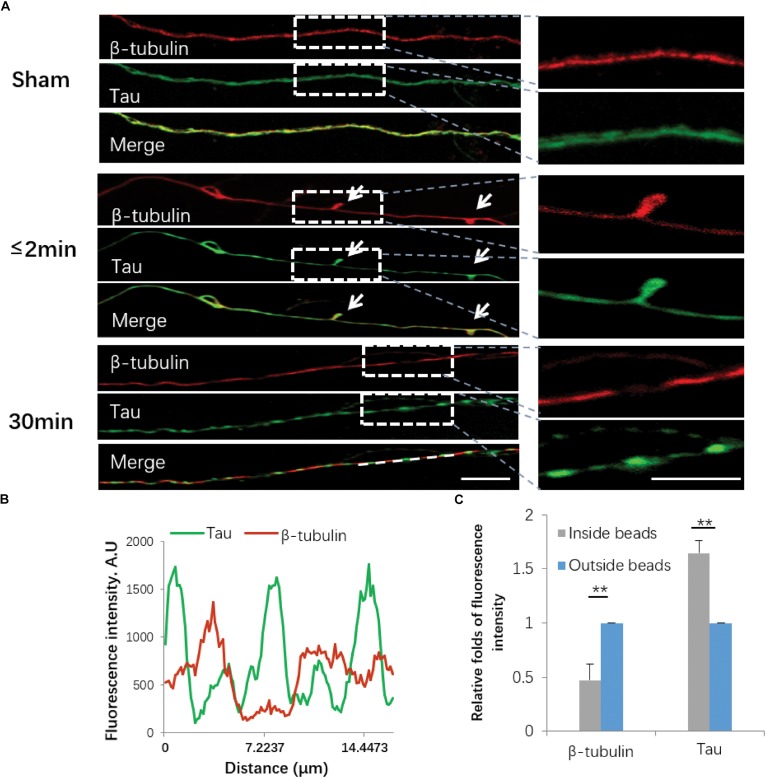
Immunofluorescence of cytoskeletal components following stretch. **(A)** Axons with or without stretch injury showed a distinctive expression pattern of both β-tubulin (red) and Tau (green). White arrows indicate immediate emergence of undulation (≤2 min) after stretch (white arrows). Representative images on the right show the white rectangle areas within the left images under high magnification. **(B)** Quantification of line scanning results within the marked area (white line). **(C)** Quantitative analysis for the fluorescence intensities of tubulin and Tau within and outside of the spheroids 30 min post-stretch (all data were presented as mean ± SE; paired-samples *t*-test, ^∗∗^*P* < 0.01; *n* = 7). Scale bar = 10 μm.

Damage to the axonal cytoskeleton is a prominent feature of axonal degeneration after traumatic axonal injury. In order to further investigate the axonal degeneration induced by our *in vitro* stretch model, we performed immunostaining of β-tubulin and Tau. For sham-operated axons, β-tubulin and Tau were almost uniformly distributed along axons. Immediately (≤2 min) after 50% stretch for 20 ms, we detected undulation of the mechanical stretch-induced cytoskeleton. Thirty minutes after stretch, the fluorescence intensity of β-tubulin and Tau in axons became notably uneven (Figures 5A,B). In beading areas, the fluorescence intensity of β-tubulin was significantly decreased (*P* < 0.01). However, the fluorescence intensity of Tau was significantly increased compared with adjacent areas within the same axon (*P* < 0.01) (Figure 5C).

### Stretch-Induced Neuronal Cell Death

**FIGURE 6:**
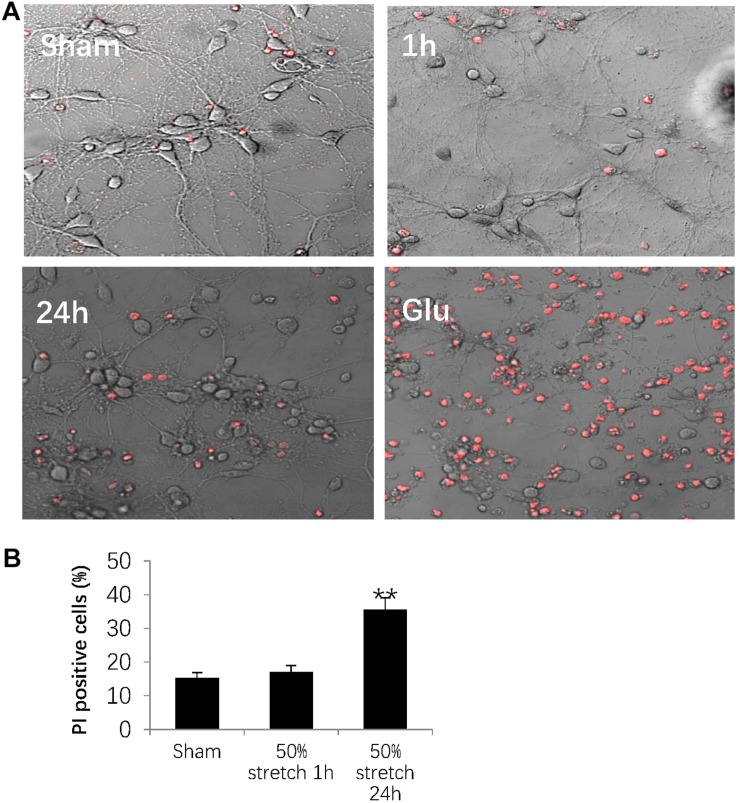
Stretch-induced neuronal cell death. **(A)** Representative images showing 50% stretch-induced neuronal cell death 1 and 24 h post-injury. Dead cells were detected by propidium iodide (PI) staining (red fluorescence), and total cell number was measured using transmitted light under confocal microscopy. The positive control was defined as a 24-h neuronal culture treated with 0.5 mM glutamate (Glu) for 30 min. **(B)** Quantitative analysis of neuronal cell death by calculating the ratio between the numbers of PI-positive cells and total cells (all data were presented as mean ± SE; one-way ANOVA, *n* = 15 in each group from three independent chambers; ^∗∗^*P* < 0.01). Scale bar = 100 μm.

In order to determine stretch-induced neuronal cell death, we used PI staining to label dead cells induced by 50% stretch for either 1 or 24 h post-stretch. The number of dead cells was then normalized against the total cell number by using transmitted light under confocal microscopy (Figure 6A). As a positive control, we assessed neuronal numbers 24 h after stretch treatment with 0.5 mM glutamate for 30 min (Figure 6A). Further quantitative analysis indicated that 24 h following stretch, there was an apparent increase in the number of PI-positive neurons from groups subjected to 50% stretch in comparison with that in other groups (Figure 6B). In summary, our results indicated that the interruption of axonal cytoskeleton integrity eventually led to neuronal death during subacute stages following AAD.

## Discussion

We created a novel *in vitro* model for DAI using elastic silicon chambers for primary neuronal cultures. Furthermore, morphological changes of axons were measured in acute phase under different mechanical parameters. It was noted in terms of the minimum strain threshold that is sufficient to result in AAD within 30 min. Optimal parameters for studying AAD was also verified with different parameters. Accordingly, a series of important pathophysiological reactions of axons were validated under the condition of injury caused by certain parameters. Therefore, in order to accurately assess pathophysiological changes of axons in the ultra-early stage of DAI, the groundwork was laid down to obtain the parameters for its systematical modeling.

We succeeded in modeling the precisely controllable strain required for axonal stretch injury via silicon chamber stretching. In terms of the uneven distribution of the strain field during chamber stretching, we selected axons that were located in a preselected central area within the axon to ensure homogeneity throughout our experiments. Notably, axons became brittle and easily ruptured only when they were subjected to rapid, as well as overwhelming, mechanical loading, which normally allows axons to accommodate mechanical deformation under certain thresholds. We specifically tested distinct sets of stretch intensity and duration. There was no obvious morphological abnormality of axons with a lower strain condition (<17.75 ± 1.65%) during the acute phase within 30 min after injury. In contrast, AAD was observed in axons following injury with a higher strain condition (>17.75 ± 1.65%), which also produced various morphological alterations, including axon beading, secondary axotomy, and a marked increase in localized Ca^2+^ influx (Morrison et al., 2011; Siedler et al., 2014).

Furthermore, localized swellings, or axonal beads along the same axons, usually leading to secondary axotomy, were observed during our experiments. In accordance with this, many previous *in vivo* studies have suggested that uniaxial tension emerging in the long-projecting tracts of the central nervous system was normally associated with the progression of varicosities, also known as localized swellings or axonal beads, in comparison with the formation of transient axonal blebs, which are normally caused by sudden shear strain (Bigler and Maxwell, 2012; Siedler et al., 2014; Maxwell et al., 2015).

Our study also showed that disruption of the integrity and continuity of the axonal cytoskeleton underlies the observed morphological alterations, inevitably leading to AAD and subsequent neuronal death. Axons are a well-connected multi-structural entirety containing different components of the axonal cytoskeleton (Montanino and Kleiven, 2018). Our results clearly revealed that when axolemmal disruption was initially triggered, Tau proteins accumulated in the forming beads, possibly due to their failure to leave the disconnected bundles of microtubules in comparison with β-tubulin, especially 30 min after stretch (Hill et al., 2016).

Following axolemmal disruption, perturbation of the ionic equilibrium leads to the intracellular accumulation of [Ca^2+^] (Morrison et al., 2011; Siedler et al., 2014), which was most evident in axonal beads in the present study. Others have demonstrated that sequestered Ca^2+^ within mitochondria disrupts oxidative metabolism by activating calcium-dependent calpains, as well as caspases among other signaling cascades, inevitably leading to cytoskeletal breakdown and eventually, neuronal death (Siedler et al., 2014; Rubiano et al., 2015). This was also evident in our present study, as indicated by PI staining 24 h after 50% stretch.

In addition, previous studies showed that *in vitro* neuronal injury occurring after 3-D compression was not only strain but also rate dependent (Bar-Kochba et al., 2016; Montanino and Kleiven, 2018); our current study also showed that neurons with larger stretch rates exhibited more severe secondary axotomy compared with those subjected to lower stretch rates. This suggests that the incidence and severity of AAD were potentially strain rate dependent when neurons were subjected to a strain that was higher than their threshold (17.75 ± 1.65%). Therefore, we established a reliable *in vitro* DAI model via uniaxial stretch using a silicon chamber under well-defined biomechanical conditions (strain_axon_ = 41.8 ± 4.4% lasting for 20 ms). Our model can be applied for *in vitro* drug screening, as well as developing new treatments for the attenuation or prevention of DAI.

### Limitation

For a long time, there is no clear standard of accurate parameters for precisely modeling DAI. In the present study, the pathophysiological changes of axons were investigated at the early stage after axonal traction injury. The parameters obtained in this study were mainly applied for establishing DAI cell models in terms of acute-stage axonal injury. Arguably, milder injury parameters (less than 17.75 ± 1.65%) are sufficient to generate DAI cell models; however, this particular approach may not necessarily lead to significant AAD. Given the fact that the growth direction of axons in the central region of the Petri dish is irregular, only a few axons (angle between axon and *x*-axis is less than or equal to 30°) can be employed for experiments through uniaxial stretch injury. Therefore, micro-patterned silica gel Petri dishes should be introduced in future investigations, which have those axons grow horizontally so as to significantly improve modeling efficiency.

## Ethics Statement

All procedures regarding usage of mice complied with the standard protocol, which was approved by the Animal Care and Use Committee of Tongji Medical School in Huazhong University of Science and Technology.

## Author Contributions

YL and JS designed the research. YL, CL, CG, KZ, and JC performed the research. YL, CL, and CG analyzed and interpreted the data. TL wrote the manuscript. JS and TL revised and approved the manuscript.

## Conflict of Interest

The authors declare that the research was conducted in the absence of any commercial or financial relationships that could be construed as a potential conflict of interest.

## Supplementary Material

FIGURE S1 Illustration of preparation for compartmental culture. Following UV sterilization, the silicon chamber was pre-coated by PLL. Sylgard 184 silicone elastomer was then applied to the central area of each chamber as a barrier, which subdivided the chamber into two left–right half parts. Six hours prior to removal of the barrier, primary neurons were plated in the chamber, and then the barriers were removed afterward.

FIGURE S2 Comparison of the morphological changes after stretch injury for axons from DIV 7 and DIV 12 neuronal cultures. **(A)** Axonal responses, from DIV7 and 12 neuronal cultures, at 30 min after stretch injury (50%, 20 ms), using transmitted light under confocal microscopy. **(B)** Quantitative analysis of the extent of AAD for axons from DIV7 and DIV12 neuronal cultures, at 30 min after stretch injury under different strain rates (all data were presented as mean ± SE; Paired-samples *t*-test, all *P* > 0.05; *n* = 6).

## References

[B1] Bar-Kochba E. Scimone M. T. Estrada J. B. Franck C. (2016). Strain and rate-dependent neuronal injury in a 3D in vitro compression model of traumatic brain injury. *Sci. Rep.* 6:30550. 10.1038/srep30550 27480807 PMC4969749

[B2] Beaudoin G. R. Lee S. H. Singh D. Yuan Y. Ng Y. G. Reichardt L. F. (2012). Culturing pyramidal neurons from the early postnatal mouse hippocampus and cortex. *Nat. Protoc.* 7 1741–1754. 10.1038/nprot.2012.099 22936216

[B3] Bigler E. D. Maxwell W. L. (2012). Neuropathology of mild traumatic brain injury: relationship to neuroimaging findings. *Brain Imaging Behav.* 6 108–136. 10.1007/s11682-011-9145-0 22434552

[B4] Dollé J. P. (2013). An organotypic uniaxial strain model using microfluidics. *Lab Chip* 13 432–442. 10.1039/c2lc41063j 23233120 PMC3546521

[B5] Gennarelli T. A. Thibault L. E. Adams J. H. Graham D. I. Thompson C. J. Marcincin R. P. (1982). Diffuse axonal injury and traumatic coma in the primate. *Ann. Neurol.* 12 564–574. 10.1002/ana.410120611 7159060

[B6] He X. S. Xiang Z. Zhou F. Fu L. A. Shuang W. (2004). Calcium overloading in traumatic axonal injury by lateral head rotation: a morphological evidence in rat model. *J. Clin. Neurosci.* 11 402–407. 10.1016/j.jocn.2004.01.001 15080957

[B7] Hill C. S. Coleman M. P. Menon D. K. (2016). Traumatic axonal injury: mechanisms and translational opportunities. *Trends Neurosci.* 39 311–324. 10.1016/j.tins.2016.03.002 27040729 PMC5405046

[B8] Hyder A. A. Wunderlich C. A. Puvanachandra P. Gururaj G. Kobusingye O. C. (2007). The impact of traumatic brain injuries: a global perspective. *Neurorehabilitation* 22 341–353. 18162698

[B9] Kilinc D. Gallo G. Barbee K. A. (2009). Mechanical membrane injury induces axonal beading through localized activation of calpain. *Exp. Neurol.* 219 553–561. 10.1016/j.expneurol.2009.07.014 19619536 PMC2747288

[B10] LaPlaca M. C. Cullen D. K. McLoughlin J. J. Cargill R. N. (2005). High rate shear strain of three-dimensional neural cell cultures: a new in vitro traumatic brain injury model. *J. Biomech.* 38 1093–1105. 10.1016/j.jbiomech.2004.05.032 15797591

[B11] Li Y. Song J. Liu X. Zhang M. An J. Sun P. (2013). High expression of STIM1 in the early stages of diffuse axonal injury. *Brain Res.* 1495 95–102. 10.1016/j.brainres.2012.12.005 23261659

[B12] Marmarou A. Foda M. A. van den Brink W. Campbell J. Kita H. Demetriadou K. (1994). A new model of diffuse brain injury in rats. part I: pathophysiology and biomechanics. *J. Neurosurg.* 80 291–300. 10.3171/jns.1994.80.2.0291 8283269

[B13] Maxwell W. L. Bartlett E. Morgan H. (2015). Wallerian degeneration in the optic nerve stretch-injury model of traumatic brain injury: a stereological analysis. *J. Neurotrauma* 32 780–790. 10.1089/neu.2014.3369 25333317

[B14] Meythaler J. M. Peduzzi J. D. Eleftheriou E. Novack T. A. (2001). Current concepts: diffuse axonal injury-associated traumatic brain injury. *Arch. Phys. Med. Rehabil.* 82 1461–1471. 10.1053/apmr.2001.25137 11588754

[B15] Montanino A. Kleiven S. (2018). Utilizing a structural mechanics approach to assess the primary effects of injury loads onto the axon and its components. *Front. Neurol.* 9:643. 10.3389/fneur.2018.00643 30127763 PMC6087765

[B16] Morrison B. R. Elkin B. S. Dolle J. P. Yarmush M. L. (2011). In vitro models of traumatic brain injury. *Annu. Rev. Biomed. Eng.* 13 91–126. 10.1146/annurev-bioeng-071910-124706 21529164

[B17] Povlishock J. T. Christman C. W. (1995). The pathobiology of traumatically induced axonal injury in animals and humans: a review of current thoughts. *J. Neurotrauma* 12 555–564. 10.1089/neu.1995.12.555 8683606

[B18] Ross D. T. Meaney D. F. Sabol M. K. Smith D. H. Gennarelli T. A. (1994). Distribution of forebrain diffuse axonal injury following inertial closed head injury in miniature swine. *Exp. Neurol.* 126 291–299. 7925827 10.1006/exnr.1994.1067

[B19] Rubiano A. M. Carney N. Chesnut R. Puyana J. C. (2015). Global neurotrauma research challenges and opportunities. *Nature* 527 S193–S197. 10.1038/nature16035 26580327

[B20] Siedler D. G. Chuah M. I. Kirkcaldie M. T. Vickers J. C. King A. E. (2014). Diffuse axonal injury in brain trauma: insights from alterations in neurofilaments. *Front. Cell. Neurosci.* 8:429. 10.3389/fncel.2014.00429 25565963 PMC4269130

[B21] Smith D. H. Hicks R. Povlishock J. T. (2013). Therapy development for diffuse axonal injury. *J. Neurotrauma* 30 307–323. 10.1089/neu.2012.2825 23252624 PMC3627407

[B22] Smith D. H. Wolf J. A. Lusardi T. A. Lee V. M. Meaney D. F. (1999). High tolerance and delayed elastic response of cultured axons to dynamic stretch injury. *J. Neurosci.* 19 4263–4269. 10.1523/jneurosci.19-11-04263.1999 10341230 PMC6782601

[B23] Staal J. A. Dickson T. C. Chung R. S. Vickers J. C. (2007). Cyclosporin-A treatment attenuates delayed cytoskeletal alterations and secondary axotomy following mild axonal stretch injury. *Dev. Neurobiol.* 67 1831–1842. 10.1002/dneu.20552 17702000

[B24] Strich S. J. (1956). Diffuse degeneration of the cerebral white matter in severe dementia following head injury. *J. Neurol. Neurosurg. Psychiatry* 19 163–185. 10.1136/jnnp.19.3.163 13357957 PMC497203

[B25] Wang H. C. Duan Z. X. Wu F. F. Xie L. Zhang H. Ma Y. B. (2010). A new rat model for diffuse axonal injury using a combination of linear acceleration and angular acceleration. *J. Neurotrauma* 27 707–719. 10.1089/neu.2009.1071 20039778

